# Mutant IDH1 confers resistance to energy stress in normal biliary cells through PFKP-induced aerobic glycolysis and AMPK activation

**DOI:** 10.1038/s41598-019-55211-w

**Published:** 2019-12-11

**Authors:** Hiroaki Fujiwara, Keisuke Tateishi, Kento Misumi, Akimasa Hayashi, Kaori Igarashi, Hiroyuki Kato, Takuma Nakatsuka, Nobumi Suzuki, Keisuke Yamamoto, Yotaro Kudo, Yoku Hayakawa, Hayato Nakagawa, Yasuo Tanaka, Hideaki Ijichi, Hirofumi Kogure, Yosuke Nakai, Hiroyuki Isayama, Kiyoshi Hasegawa, Masashi Fukayama, Tomoyoshi Soga, Kazuhiko Koike

**Affiliations:** 1 Department of Gastroenterology, Graduate School of Medicine, The University of Tokyo, 7-3-1 Hongo, Bunkyo-ku, Tokyo, 113-8655 Japan; 2 Division of Gastroenterology, The Institute for Adult Diseases, Asahi Life Foundation, 2-2-6 Bakurocho, Chuo-ku, Tokyo, 103-0002 Japan; 3 Department of Pathology, Graduate School of Medicine, The University of Tokyo, 7-3-1 Hongo, Bunkyo-ku, Tokyo, 113-8655 Japan; 4 Institute for Advanced Biosciences, Keio University, 246-2 Kakuganji, Tsuruoka, Yamagata, 997-0052 Japan; 5 Hepato-Biliary-Pancreatic Division, Department of Surgery, Graduate School of Medicine, The University of Tokyo, 7-3-1 Hongo, Bunkyo-ku, Tokyo, 113-8655 Japan; 6 Department of Gastroenterology, Graduate School of Medicine, Juntendo University, 2-1-1 Hongo, Bunkyo-ku, Tokyo, 113-8421 Japan

**Keywords:** Cancer metabolism, Mechanisms of disease

## Abstract

Metabolism is a critical regulator of cell fate determination. Recently, the significance of metabolic reprogramming in environmental adaptation during tumorigenesis has attracted much attention in cancer research. Recurrent mutations in the isocitrate dehydrogenase (IDH) 1 or 2 genes have been identified in several cancers, including intrahepatic cholangiocarcinoma (ICC). Mutant IDHs convert α-ketoglutarate (α-KG) to 2-hydroxyglutarate (2-HG), which affects the activity of multiple α-KG-dependent dioxygenases including histone lysine demethylases. Although mutant *IDH* can be detected even in the early stages of neoplasia, how IDH mutations function as oncogenic drivers remains unclear. In this study, we aimed to address the biological effects of *IDH1* mutation using intrahepatic biliary organoids (IBOs). We demonstrated that mutant IDH1 increased the formation of IBOs as well as accelerated glucose metabolism. Gene expression analysis and ChIP results revealed the upregulation of platelet isoform of phosphofructokinase-1 (PFKP), which is a rate-limiting glycolytic enzyme, through the alteration of histone modification. Knockdown of the *Pfkp* gene alleviated the mutant IDH1-induced increase in IBO formation. Notably, the high expression of PFKP was observed more frequently in patients with IDH-mutant ICC compared to in those with wild-type IDH (p < 0.01, 80.9% vs. 42.5%, respectively). Furthermore, IBOs expressing mutant IDH1 survived the suppression of ATP production caused by growth factor depletion and matrix detachment by retaining high ATP levels through 5ʹ adenosine monophosphate-activated protein kinase (AMPK) activation. Our findings provide a systematic understanding as to how mutant IDH induces tumorigenic preconditioning by metabolic rewiring in intrahepatic cholangiocytes.

## Introduction

Intrahepatic cholangiocarcinoma (ICC) is the second most common liver cancer with an overall poor prognosis^1^. Due to the limited effectiveness of drugs, the five-year survival rate remains less than 10%^2^. Primary risk factors of ICC include: infection by liver flukes or hepatitis virus, primary sclerosing cholangitis, and intrahepatic stones^3^. Although whole genomic sequencing has identified the mutational landscape of ICC^4,5^, its pathogenesis remains unclear.

The mutations in isocitrate dehydrogenase 1 (IDH1) or isocitrate dehydrogenase 2 (IDH2) genes are identified in 10–30% of ICC. Specifically, the mutations are found in ICC without the infection of liver flukes or hepatitis virus^6,7^. They are often mutually exclusive with mutations in Kirsten rat sarcoma viral oncogene homolog (*KRAS*) and tumor protein p53 (*TP53*) genes^8,9^. ICC with IDH mutation have specific features such as DNA hypermethylation or distinct drug sensitivity^10–12^. Typically, IDH is a metabolic enzyme that catalyzes the oxidative decarboxylation of isocitrate to α-ketoglutarate (α-KG) and CO_2_ during the citric acid cycle^11^. In mutant IDH, a specific metabolite R(–)-2-hydroxyglutarate (2-HG) is produced from α-KG^12^. 2-HG inhibits the family of α-KG–dependent dioxygenase enzymes, including lysine histone demethylases (KDM) and the ten-eleven translocation (TET) family of DNA hydroxylases^13,14^. The aberrant epigenetic regulation caused by IDH mutation is associated with abnormal cellular differentiation and/or carcinogenesis^15,16^.

Mutant IDH was reported to affect the initiation of Grade II/III gliomas or acute myeloid leukemia^15–17^. Although ICC originates from several cell lineages including hepatocytes, cholangiocytes or, liver progenitor cells^18,19^, how IDH mutation contributes to oncogenic processes of ICC is still unknown. A recent study showed that mutant IDH inhibits hepatocyte nuclear factor 4-alpha (HNF-4α) expression to preferentially induce biliary lineage cells from liver progenitors, resulting in ICC formation combined with *Kras* mutations in mice^20^. Recently, organoid techniques have made it possible to culture normal epithelial cells derived from primary tissues^21–23^. In this study, we established mouse intrahepatic biliary organoids (IBOs) that expressed mutant IDH1 to elucidate the functional role of mutant IDH1 in biliary tumorigenesis.

## Results

### IDH1 mutation enhances the formation of biliary organoids established from murine liver

**Figure 1:**
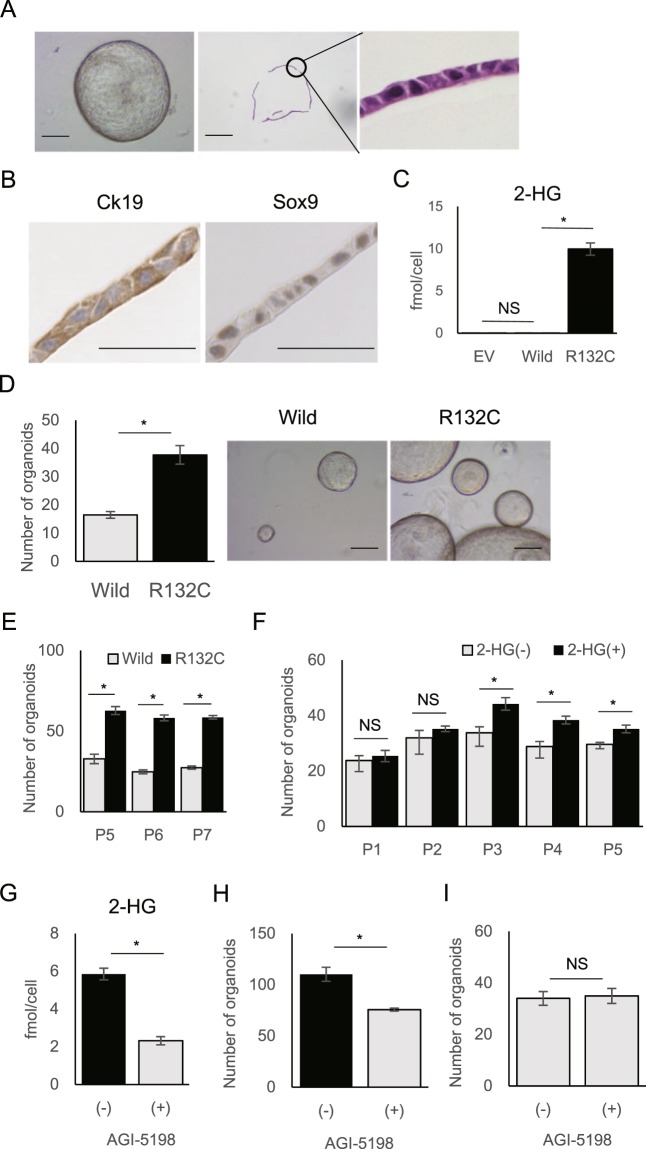
*IDH1* mutation enhances the formation of biliary organoids established from murine liver. (**A**) The left panel shows a representative image of IBOs from wild-type mice (8-weeks old, male) at day 7. The middle and left panels show hematoxylin and eosin (HE) staining of them. Scale bars, 250 μm (middle and left panels) and 25 μm (right panel). (**B**) Immunohistochemical staining of Ck19 and Sox9 in IBOs. Scale bars, 20 μm. (**C**) The amount of 2-HG measured by CE-MS in cell extracts from IBOs stably expressing empty vector (EV), wild-type (Wild), and mutant IDH1 (R132C). (n = 4, *P < 0.05, NS not significant). (**D**) The number of IBOs (>100 μm) at 7 d after plating (n = 4, 3,000 cells per group, *P < 0.05) and their representative images. Scale bars, 250 μm. The assay was performed at passage 4. (**E**) Organoid-forming efficiency of the indicated IBOs during serial passage (P5–7) after puromycin selection. The number of organoids (>100 μm) at 7 d after plating (n = 4, 3,000 cells per group, *P < 0.05). (F) IBOs established from wild-type mice treated with 10 mM 2-HG ( + ) or vehicle (−) upon serial passage. The number of organoids (>100 μm) at 7 d after plating (n = 5, 3,000 cells per group, *P < 0.05, NS not significant). (**G**) 2-HG levels of mut-IBOs treated with 20 μM AGI-5198 (+) or DMSO vehicle (−). (**H,I**) The number (>100 μm) of mut-IBOs (**H**) and wt-IBOs (**I**) treated with 20 μM AGI-5198 (+) or DMSO vehicle at 7 d after plating (n = 4, 10,000 cells per group, *P < 0.05).

To elucidate how mutant IDH affects the molecular or biological features of normal intrahepatic cholangiocytes, we established IBOs from murine normal liver cells (Fig. 1A and Supplementary Fig. 1A)^24^. Given that organoids maintained the characteristics of primary cells^24^, we applied this system to estimate the biological and metabolic traits triggered by *IDH1* mutation in normal biliary epithelial cells. IBOs were composed of a monolayer of biliary lineage cells expressing biliary marker genes, including cytokeratin 7 (*Ck7*), cytokeratin 19 (*Ck19*), and hepatocyte nuclear factor 1-beta (*Hnf1β*), but not hepatocyte marker genes such as transthyretin (*Ttr*) and aldolase B (*AldoB*) (Fig. 1A and Supplementary Fig. 1A). Immunohistochemistry confirmed the expression of CK19 and SRY (sex determining region Y)-box 9 (SOX9) as biliary marker proteins in IBOs (Fig. 1B). The established IBOs were transduced with lentiviral vectors expressing human wild-type or mutant IDH1 (R132C) (Supplementary Fig. 1B). The activity of transduced mutated IDH1 was confirmed by production of 2-HG (Fig. 1C). We found that IBOs expressing mutant IDH1 (mut-IBOs) exhibited enhanced organoid-forming efficiency compared to IBOs expressing wild-type IDH1 (wt-IBOs), constitutively through serial passages (Fig. 1D, E). The 2-HG addition also enhanced the formation of IBOs after the third passage (Fig. 1F). In addition, a specific inhibitor of mutant IDH1^25^, AGI-5198, significantly reduced the 2-HG production and hampered the formation of mut-IBOs. The effects were specific for mut-IBOs because AGI-5198 did not affect the growth of wt-IBOs (Fig. 1G,I). These results indicated that mutant IDH1 has the biological potential to enhance the IBO forming ability.

### Mutant IDH1 upregulates glucose metabolism in IBOs

**Figure 2:**
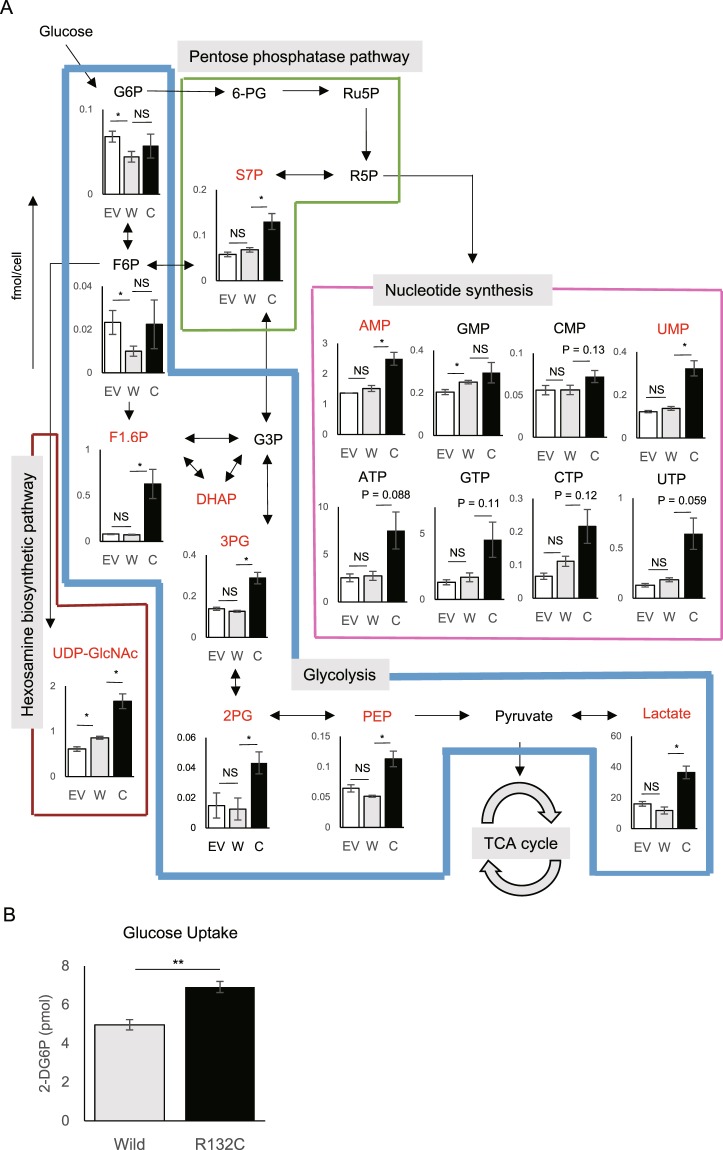
Mutant IDH1 increases glycolytic flux in IBOs. (**A**) Metabolome analysis of IBOs stably expressing empty vector (EV), wild-type IDH1 (Wild) or mutant IDH1 (R132C) by CE-MS (n = 4, *P < 0.05, NS not significant). (**B**) Glucose uptake in the indicated IBOs (n = 3, *P < 0.05). Abbreviation; α-KG, α-ketoglutarate; AMP, adenosine monophosphate; ATP, adenosine triphosphate; CMP, cytidine monophosphate; CTP, cytidine triphosphate; DHAP, dihydroxyacetone phosphate; F1,6 P, fructose-1,6-bisphosphate; F6P, fructose 6-phosphate; GMP, guanosine monophosphate; G3P, glyceraldehyde 3-phosphate; G6P, glucose-6-phosphate; GTP, guanosine triphosphate; 6-PG, 6-phosphogluconate; PEP, phosphoenolpyruvate; 3PG, 3-phosphoglycerate; 2PG, 2-phosphoglycerate; R5P, ribose 5-phosphate; Ru5P, ribulose 5-phosphate; S7P, sedoheptulose-7-phosphate; UDP, uridine diphosphate UDP-GlcNAc, uridine diphosphate N-acetylglucosamine; UMP, uridine monophosphate; UTP, uridine triphosphate.

**Figure 3:**
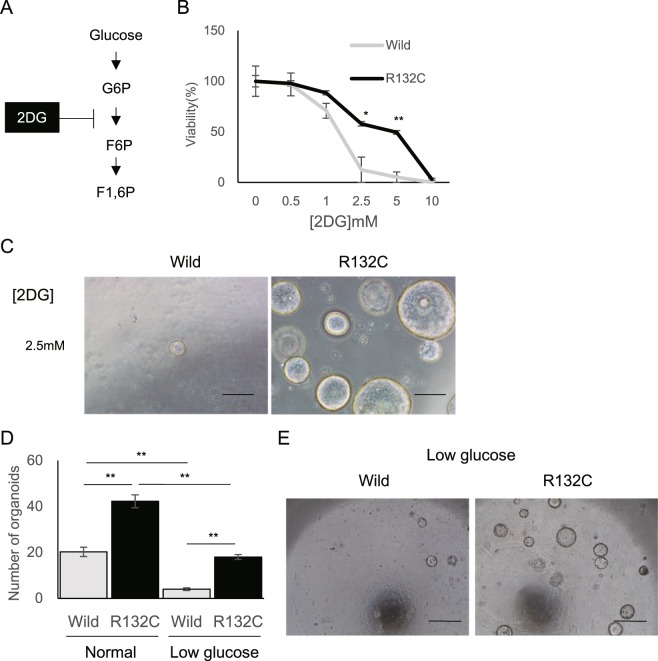
The glycolytic inhibitor 2-DG or glucose restriction hampers the formation of IBOs, and mut-IBOs were resistant to the inhibition. (**A**) 2-DG competitively inhibits the conversion of G6P to F6P in glycolysis. (**B**) The number of organoids (>100 μm) at 5 d after plating in medium with or without 2DG (normalized to that in the 2DG-free medium, n = 4, 5,000 cells per group *P < 0.05). (**C**) Representative images. Scale bars, 250 μm. (**D**) The number of IBOs (>100 μm) was counted at 7 d after plating under the normal culture condition (Normal) and the low glucose condition (Low glucose), replacing advanced-DMEM/F12 (3 g/L glucose) to L0091, glucose-free DMEM/F12 instead (n = 4, 5,000 cells per group, **P < 0.01). Low glucose medium contains a little glucose derived from Wnt3a- and RSPO1-conditioned medium; the final concentration of glucose is 0.2 g/L. (**E**) Representative images. Scale bars, 500 μm.

To uncover metabolic traits underlying the increased forming ability in mut-IBOs, metabolic profiles of the IBOs were analyzed by capillary electrophoresis-mass spectrometry (CE-MS) (Fig. 2A and Supplementary Fig. 2). Notably, mut-IBOs displayed enrichment of glycolytic intermediates including fructose-1,6-bisphosphate (F1,6 P), 3-phosphoglycerate (3PG), 2-phosphoglycerate (2PG), phosphoenolpyruvate (PEP), and lactate compared with wt-IBO (Fig. 2A). In addition, the metabolites of other glucose-utilizing pathways sedoheptulose-7-phosphate (S7P), adenosine monophosphate (AMP), uridine monophosphate (UMP) in the pentose phosphate pathway (PPP), and UDP-N-acetylglucosamine in the hexosamine biosynthesis pathway (HBP), were significantly accumulated in mut-IBOs (Fig. 2A). Consistent with an increase in glycolysis, glucose uptake was significantly enhanced in mut-IBOs compared to wt-IBOs (Fig. 2B). Given that all of these metabolic profiles were identified under aerobic culture conditions with sufficient glucose and glutamine, such preferred aerobic glycolysis seemed to be similar to the ‘Warburg effect’ - a well-known hallmark of rapidly proliferating mammalian cells^26^. However, some metabolites in the TCA cycle, including citrate, fumarate, and malate, were also elevated in mut-IBOs (Supplementary Fig. 2), indicating that mutations in IDH1 lead to higher glucose metabolism. Since reprogramming from differentiated cells to cancer cells is often accompanied by aerobic glycolytic phenotypes, we aimed to investigate if activated glycolysis correlates with the enhanced formation of mut-IBOs by treatment with a glycolytic inhibitor, 2-deoxy-d-glucose (2DG) (Fig. 3A). Although 2DG reduced the organoid-forming efficiency of both wt- and mut-IBOs in a dose-dependent manner, the formation of mut-IBOs was relatively resistant to the inhibitory effects of 2DG compared to wt-IBOs (Fig. 3B,C). The forming capacity of mut-IBOs compared to wt-IBOs was relatively conserved under a low glucose condition (Fig. 3D,E). These results suggest that the formation of IBOs depends on glucose as a critical energy source, and IDH1 mutation allows for IBOs to become resistant to glucose restriction.

### Mutant IDH1 activates glycolysis through the upregulation of Pfkp gene

**Figure 4:**
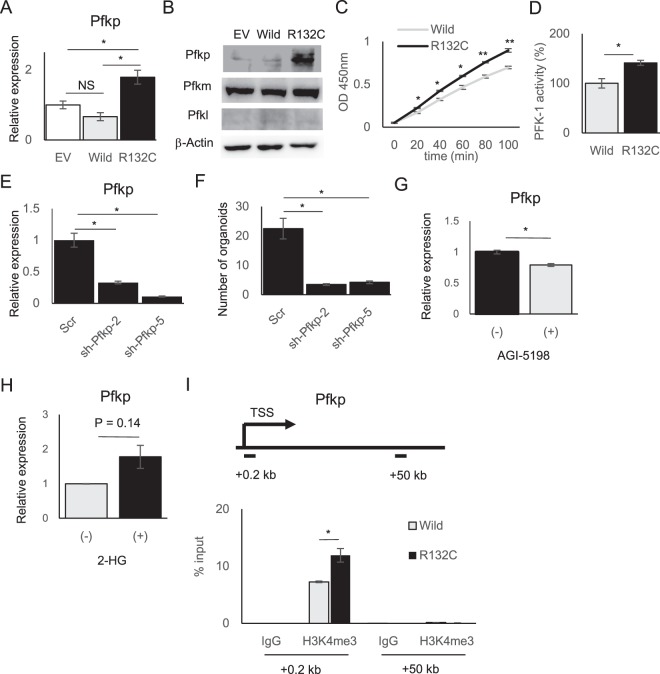
Mutant IDH1 activates glycolysis in IBOs through the upregulation of *Pfkp*. (**A**) The level of *Pfkp* expression in the indicated IBOs by RT-qPCR (n = 4, *P < 0.05, NS not significant). (**B**) Protein expression levels of Pfkp, Pfkm, and Pfkl as determined by western blotting. (**C,D**) Enzymatic assay of PFK-1 activity in the indicated IBOs. Time course of optical absorbance at 450 nm in (**C**), and comparison of PFK-1 activity in (**D**) (n = 4, *P < 0.05, **P < 0.01). (**E**) Gene expression of *Pfkp* in mut-IBOs transfected with shRNA for Pfkp (shPfkp-2, 5) or scrambled shRNA (Scr) as control (n = 4, *P < 0.05). (**F**) Organoid-forming efficiency of the indicated IBOs (>100 μm) at 7 days after plating (n = 4, 3000 cells per group, *P < 0.05). (**G**) Gene expression of *Pfkp* in mut-IBOs treated with 20 μM AGI-5198 (+) or DMSO vehicle (−) (n = 4, *P < 0.05). (**H**) IBOs established from wild-type mice were treated with 10 mM 2-HG (+) or vehicle (−) upon serial passage. Gene expression of *Pfkp* at passage 4 by RT-qPCR (n = 3, P = 0.14). (**I**) The level of trimethylation of histone H3 lysine4 (H3K4me3) on the promoter region of *Pfkp* by ChIP analysis in IBOs. The upper panel shows the schematic diagram of the transcriptional start site (TSS) and amplicons (0.2 kb and 50 kb downstream of the TSS). The lower graph shows ChIP- qPCR data (relative to input DNA, n = 4, *P < 0.05).

**Figure 5:**
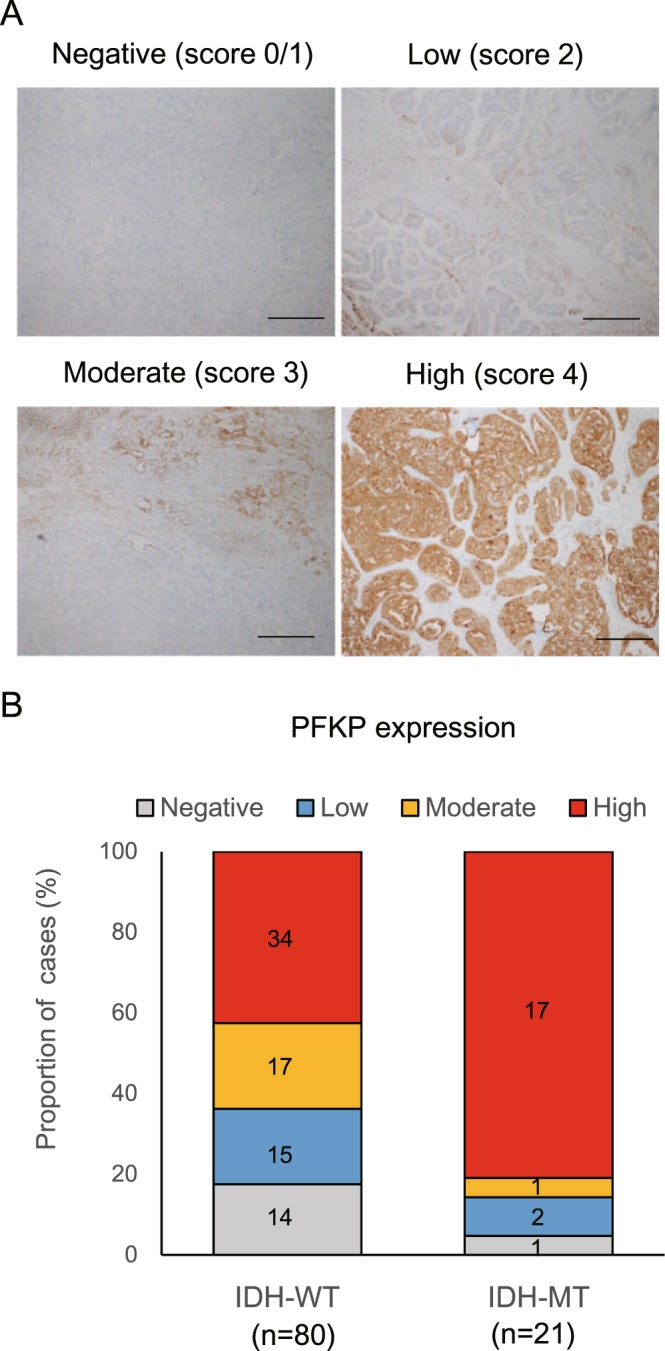
PFKP shows a high expression in IDH-mutant ICCs. (**A**) Representative images of PFKP-staining of human ICC surgical specimens. Scale bars, 500 μm. (**B**) PFKP protein expression in human ICC surgical specimens and association with IDH mutation status.

To address the molecular mechanisms underlying increased glycolysis in mut-IBO, we performed microarray gene expression analysis involving >40,000 oligonucleotide probes. Regardless of the drastic changes in organoid-forming efficiency and metabolism by IDH1 mutation, the analysis showed that only 19 genes were upregulated >2-fold, and 27 genes were downregulated <2-fold in mut-IBOs compared to wt-IBOs (P < 0.05, Supplementary Fig. 3A and Supplementary Table 1). Consistent with the effects of IDH1 mutation in terms of catalytic activities of αKG-dependent histone lysine demethylases, the levels of global histone H3 lysine 4 trimethylation (H3K4me3) and lysine 27 trimethylation (H3K27me3) were elevated in mut-IBOs, compared to wt-IBOs (Supplementary Fig. 3B) as previously reported in cancer cells^15,16^. Expression of genes encoding enzymes such as Jarid1a, Jarid1b, or Jmjd3 were not affected (Supplementary Fig. 3C). In contrast, there were no significant differences in the levels of 5-hydroxymethylcytosine (5-hmC), 5-methylcytosine (5-mC), or in the expression of Tet family genes encoding DNA demethylase (Supplementary Fig. 3C–E). In addition, we analyzed the effects on chromatin structure caused by mutant IDH1 using an assay for transposase-accessible chromatin using sequencing (ATAC-seq)^27^. Although different peak patterns were detected in certain locations, there were no significant differences in specific motif sequences binding transcription factors (TFs) between wt- and mut-IBOs (Supplementary Fig. 4A–C). These results appear to be compatible with the lack of differences in DNA methylation, and the limited number of differentially expressed genes between wt- and mut-IBOs. Notably, however, we found a significant upregulation in the expression of the platelet isoform of phosphofructokinase-1 (*Pfkp*) gene in mut-IBOs (Fig. 4A). Mammalian phosphofructokinase-1 (PFK-1) is a tetramer consisting of three isoforms; Pfkl, Pfkm, and Pfkp^28^ and catalyzes the conversion of fructose 6-phosphate (F6P) to F1,6 P, which is a rate-limiting step in the glycolytic pathway. Intriguingly, the promotion of glycolysis in mut-IBOs occurred immediately downstream the transition of F6P to F1,6 P (Fig. 2A). This led us to hypothesize that IDH1 mutation drives glycolysis through the upregulation of Pfk-1 activity. Supporting our hypothesis, we confirmed not only increases in *Pfkp* mRNA and protein levels but also identified elevated Pfk-1 activity in mut-IBOs (Fig. 4A–D). To analyze if PFKP contributes to IBO formation, we established two distinct mut-IBO lines in which *Pfkp* was stably knocked-down (Fig. 4E). Both mut-IBO lines decreased the efficiency of IBO formation by mutant IDH1 (Fig. 4F), emphasizing the notion that *IDH1* mutation promotes IBO formation by enhancing glycolysis via upregulation of PFKP. To examine the dysregulation of *Pfkp* directly caused by *IDH1* mutation, the effect of the inhibitor AGI-5198 on the expression of the *Pfkp* gene was analyzed^25^. AGI-5198 decreased the expression of *Pfkp* in mut-IBOs (Fig. 4G), and, in turn, 2-HG treatment increased the expression of *Pfkp* in IBOs from normal mouse liver (Fig. 4H), suggesting that the upregulation of *Pfkp* was attributed to *IDH1* mutation. In addition, although ATAC-seq data showed comparable accessibility surrounding the *Pfkp* locus, the level of H3K4me3 as an active transcriptional mark was significantly increased on the promoter region of *Pfkp* in mut-IBOs compared to wt-IBOs (Supplementary Fig. 4D and Fig. 4I), indicating that the upregulation of *Pfkp* was mediated by aberrant histone modification. These data indicated that the epigenetic disturbance induced by *IDH1* mutation was focally limited to specific targets, such as *Pfkp* in normal biliary cells. Furthermore, we investigated the expression status of PFKP protein in 101 surgically resected ICC specimens from our hospital. PFKP expression was detected in 69 cases (68%) (Fig. 5A,B), and the expression levels were significantly higher in ICCs harboring IDH mutations (P < 0.01, Fischer’s exact test) (Fig. 5B). Considering the significance of PFKP for growth advantage in many human cancers^29,30^, PFKP could serve as a novel metabolic target for IDH1-mutant ICCs.

### IDH1 mutation provides EGF- and HGF-independent survival in IBOs

**Figure 6:**
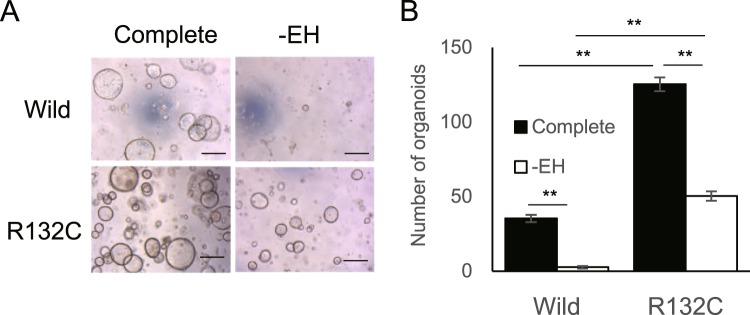
IDH1 mutation overrides the dependency on EGF and HGF in IBOs. (**A**) Representative images of IBOs at 5 d after plating (10,000 cells per well), in the presence (Complete) or absence (-EH) of EGF and HGF. Scale bars, 500 μm. (**B**) The number of organoids (>100 μm) in each group (n = 3, **P < 0.01).

We sought to assess the biological consequences of mutant IDH1-induced glycolytic activation. Consistent with previous reports showing that mutant IDH1 alone is insufficient for transformation in several cell linages^16,20^, subcutaneous inoculation of either wt-IBO or mut-IBO into nude mice failed to generate tumors (2,000,000 cells per group, n = 6, data not shown). The organoid culture systems are applied to the assessment of extracellular factor-dependency^31^. Based on the findings that cultured organoids gradually become independent of growth factors followed by oncogenic mutations^31^, we evaluated the effect of *IDH1* mutation for growth factor-dependency in the survival of IBOs. Depletion of EGF, FGF10, HGF, or R-spondin-1 significantly reduced the organoid-forming efficiency of wt-IBO (Supplementary Fig. 5A,B). Although the efficiency of mut-IBO formation decreased in the absence of EGF, FGF10, HGF, or R-spondin-1, the higher organoid-forming efficiency compared to wt-IBO was still observed (Supplementary Fig. 5A,B). Remarkably, double depletion of EGF and HGF (EH-depletion), completely blunted the formation of wt-IBOs, whereas mut-IBOs persisted (Fig. 6A,B).

### Mutant IDH1-induced AMPK activation makes IBOs override the EGF and HGF dependency

**Figure 7:**
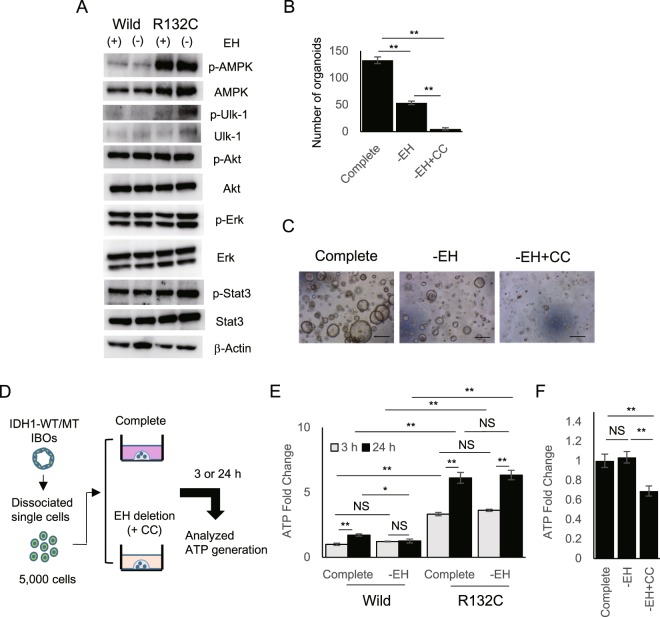
Mutant IDH1-induced growth factor independence is supported by AMPK activation. (**A**) Immunoblots of AMPK, phosphorylated AMPK (p-AMPK), Ulk1, phosphorylated-Ulk1 (p-Ulk1), Erk, phosphorylated Erk (p-Erk), Akt, phosphorylated Akt (p-Akt), Stat3 and phosphorylated Stat3 (p-Stat3), in IBOs. (**B**) The number of mut-IBOs (>100 μm) at 5 d after plating (n = 5, 10,000 cells per group, **P < 0.01). (**C**) Representative images. Scale bars, 500 μm. (**D**) Schema of experiments analyzing ATP production in dissociated IBO cells. (**E**) ATP levels of dissociated IBO cells at 24 h after plating (5,000 cells per group, n = 4, *P < 0.05, **P < 0.01, NS not significant). (**F**) ATP levels of dissociated mut-IBO cells at 24 h after plating (5,000 cells per group), in the presence (Complete) or absence (-EH) of EGF and HGF, treated with vehicle or 5 μM Compound C (CC) (n = 5, **P < 0.01, NS not significant).

To address the molecular mechanisms underlying the sustained survival after EH-depletion, we examined the status of signal transduction pathways related to cell survival (Fig. 7A and Supplementary Fig. 6). The levels of phosphorylation of Erk, Akt, or Stat3 were comparable between wt-IBOs and mut-IBOs (Fig. 7A). In addition, these phosphorylation levels were not affected by EH-depletion (Fig. 7A). In contrast, we identified enhanced phosphorylation of AMP-activated protein kinase (AMPK) in mut-IBOs compared to wt-IBOs, regardless of EH-depletion (Fig. 7A). The two primary upstream regulators of AMPK, Lkb1, and Camkk2 were not differently activated between mut-IBOs and wt-IBOs (Supplementary Fig. 7). Intriguingly, among the multiple downstream targets of the AMPK signaling pathway^32^, Unc-51 like kinase-1 (Ulk1), which is a crucial initiator of autophagy^33^, was activated by EH-depletion only in mut-IBOs (Fig. 7A and Supplementary Fig. 7). To address whether AMPK activation contributed to the sustained formation of IBOs under EH-depletion, mut-IBOs were treated with the AMPK inhibitor, Compound C^34^. The organoid-forming capacity after EH-depletion in mut-IBOs was entirely inhibited by Compound C (Fig. 7B,C), suggesting that AMPK contributes to the growth-factor independence in cooperation with IDH1 mutation. Given that 5-aminoimidazole-4-carboxamide-1-β-D-ribofuranoside (AICAR), which is a well-known AMP analog that activates AMPK, inhibited the formation of wt-IBOs (Supplementary Fig. 8). Thus, the role of AMPK seems to be context-dependent, as previously reported^35^.

It is well-known that growth factor deprivation, as well as detachment from the extracellular matrix (ECM), induces ATP loss and subsequent cell death in normal epithelial cells^36^. When organoids are passaged as dissociated single cells, they must be exposed to energy stress without growth factors or ECM. Supporting this notion, wt-IBOs were not formed in the settings without matrigel after passage procedures. Thus, we compared the levels of cellular ATP in dissociated IBO cells just after the passage procedure (Fig. 7D,E). Mounting of the dissociated wt-IBO cells on matrigel with complete medium after passage recovered ATP production in a time-dependent manner, but EH-depletion from medium inhibited the recovery (Fig. 7E). In contrast, the dissociated mut-IBO cells restored the high levels of ATP after passage, even under EH-depletion (Fig. 7E). Given the function of AMPK in replenishing ATP after the loss of ECM attachment^36^, we considered that AMPK activation contributed to ATP production in dissociated mut-IBO cells. Consistently, AMPK inhibition significantly decreased ATP levels in the dissociated mut-IBO cells (Fig. 7F). These data supported the notion that AMPK activity contributes to ATP production under EH-depletion in mut-IBOs.

## Discussion

In this study, we found a unique metabolic status in the IDH- mutant biliary cells. Several previous reports have described the phenotype of IDH-mutant cancer cells related to hypoxia-inducible factor 1 (HIF-1) activity. For example, the survival and proliferation of IDH-mutant glioma cells were promoted by the Warburg effect due to the stabilization of HIF-1α^37^. In contrast, other studies have shown that IDH mutations downregulate the HIF-1 pathway and induce limited glycolysis, contributing to the slower growth and better prognosis in glioma cells^38^. Although we do not entirely exclude the possible involvement of Hif-1α in the IDH- mutant biliary cells, the expression of Hif-1α protein and the majority of its downstream glycolytic genes were not markedly upregulated in mut-IBOs (Supplementary Fig. 9). Upregulated HIF-1α expression by *IDH1* mutation reduced the energy flux through the TCA cycle and increased the clonogenicity of normal human astrocytes due to the activation of pyruvate dehydrogenase kinases (PDKs)^39^. In contrast, levels of some metabolites in the TCA cycle, including citrate, fumarate, and malate were elevated in mut-IBOs (Supplementary Fig. 2). Additionally, decreased levels of glutamate identified in glioma or astrocytes harboring *IDH1* mutation^40^ were not observed in the case of mut-IBOs (Supplementary Fig. 2). These findings propose a possibility that metabolic rewiring traits triggered by IDH mutation are different among distinct cell lineages. Furthermore, while mutant IDH in patient material of glioma and glioblastoma was associated with a non-Warburg phenotype in comparison with wild-type IDH tumors, the artificial introduction of mutant IDH into glioblastoma cell lines resulted in aerobic glycolysis^41–43^. Thus, the metabolic effects of mutant IDH can also vary between patient-derived materials and those established experimentally. To assess the possibility of clinical trials focusing on the metabolic traits of mutant IDH tumors as in gliomas^44^, the metabolome analysis of clinical samples of ICC would be needed in the near future.

It was noteworthy that there was a difference in the organoid-forming efficiency between mut-IBOs and wt-IBOs treated with 2-HG (Fig. 1D–F). We speculate two possibilities for the different extent of growth promotion. The first is a possibility that the intracellular concentration of 2-HG in wt-IBO cells treated with 2-HG was lower than in mut-IBOs due to the limited cell permeability of R-2-HG or the run-in effect^17^. The second is a possible 2-HG independent function of mutant IDH1, which might be supported by a previous paper showing that DNA methylation profiles of 2-HG treated cells could not entirely recapitulate those of mutant IDH1 cells^45^.

In general, as the increasing level of AMP mirrors the decrease in ATP under metabolic stress conditions in normal cells^32^, the metabolic situation caused by IDH1 mutation seems to be extraordinary, where both AMP and ATP accumulated. Similarly, the activation of AMPK in mut-IBOs was rather paradoxical in terms of its energy sensor function^32^ because ATP levels remained unperturbed in mut-IBOs (Fig. 2). As the AMP-induced activation of AMPK is allosterically much more potent than the inhibitory action of ATP based on structural research^46^, AMPK activation in mut-IBOs was likely to be induced by AMP accumulation (Fig. 2).

Despite the tumor-suppressive roles of AMPK in many cancers, recent studies have shown the pleiotropic properties of AMPK for tumor cell survival under metabolic stress^35^. In terms of the relationships of AMPK and IDH mutation, previous reports demonstrated the metabolic vulnerability of mutant IDH gliomas or glioblastomas to biguanides, such as metformin and phenformin, which activate AMPK by inhibiting mitochondrial respiratory chain complex 1^47–49^. Therefore, our findings emphasize the notion that the role of AMPK seems to be pleiotropic and context-dependent^35^.

**Figure 8:**
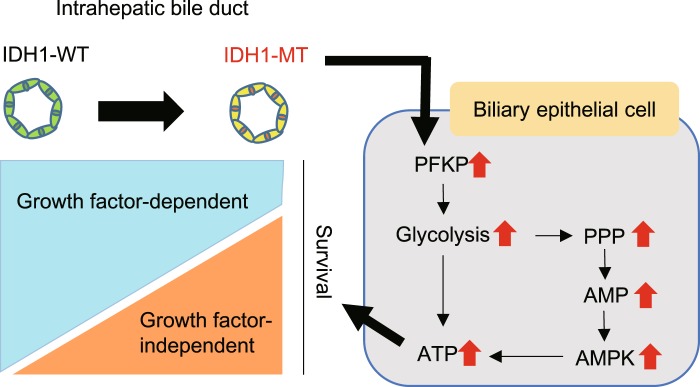
Schematic model of mutant IDH1-induced acquisition of growth factor independence via PFKP upregulation and AMPK activation in intrahepatic biliary epithelial cells.

Finally, we identified a two-way metabolic rewiring status; PFKP upregulation and AMPK activation induced by *IDH1* mutation in normal biliary cells (Fig. 8). Nonetheless, it is still necessary to elucidate the whole mechanism acquired by *IDH1* mutation that finally accomplishes biliary tumor development.

## Methods

### Liver organoid culture

Organoids derived from murine liver were established according to published protocols^24^. Resected mouse liver was minced under sterile conditions, washed in Hank’s balanced salt solution without Ca 2^+^ and Mg 2^+^ (HBSS-) containing 10 mM HEPES, 0.5 mM EDTA, and then naturally sedimented. After removal of the supernatant, the minced liver was digested in HBSS+ containing 10 mM HEPES, 1 mg/mL of collagenase type 4 (Invitrogen, Carlsbad, CA) and 0.5 mg/mL of pronase (Sigma-Aldrich, St. Louis, MO, USA) for 10–15 min. The suspension was filtered through cell strainers (100 μm) and sedimented by centrifugation (700 × *g*) for 3 min. After hemolytic treatment, the pellet was mixed with Matrigel (BD Bioscience, San Jose, CA) and cultured as previously described^24^. Culture medium was based on Advanced DMEM/F12 (Invitrogen) supplemented with B27 (Invitrogen), N2 (InvivoGen, San Diego, CA), 1.25 μM N-acetylcysteine (Wako, Osaka, Japan), 50 ng/mL EGF (Wako), 100 ng/mL FGF10 (Wako), 50 ng/mL HGF (R&D Systems, Minneapolis, MN), 10% RSPO1-conditioned medium (kindly gifted from C. Kuo and prepared as previously reported^23^), 10 nM gastrin (Sigma-Aldrich), and 10 mM nicotinamide (Sigma-Aldrich). For the first 4 d after seeding, the cells were also supplemented with 100 ng/mL Noggin (PeproTech, Rocky Hill, NJ) and 10% Wnt3a-conditioned medium (prepared as previously reported^22^). Next, 1 week after seeding, organoids were mechanically dissociated and transferred to fresh gels.

### Construction of IDH1 mutation and lentiviral infection

Human wild-type IDH1 expression vector (plasmid pFN21AE0433) was purchased from Kazusa DNA Research Institute (Chiba, Japan). The IDH1 R132C mutant was generated from the wild-type IDH1 open reading frame of this plasmid using the QuikChange Site-Directed Mutagenesis Kit (Agilent Technologies, Santa Rosa, CA) and primers 5′-GATCCCCATAAGCATGACAACCTATGATGATAGGTTC-3′ for sense and 5′- GAACCTATCATCATAGGTTGTCATGCTTATGGGGATC-3′ for antisense. The wild-type and mutant IDH1 were ligated into the multiple cloning sites of pLVSIN-EF1α Vector (TAKARA, Shiga, Japan) and these lentiviral vectors were transfected into 293 T/17 cells. Organoids were infected with concentrated lentivirus supernatant for 9–12 h and selected with puromycin (2 μg/mL).

Transduction was validated by reverse transcriptase-polymerase chain reaction (RT-PCR) using specific primers for mutant IDH1 (R132C): 5′-GGGTAGAACCTATCATCATAGGTT-3′, 5′-TCCAGGCCCAGGAACAACAA-3′, and wild-type IDH1: 5′- GGGTAGAACCTATCATCATAGGTC-3′, 5′- TCCAGGCCCAGGAACAACAA-3′.

### shRNA

Lentiviral (pLKO.1) *Pfkp* shRNA vectors TRCN0000025916 and TRCN0000025962 were purchased from Dharmacon Inc. (Lafayette, CO). and used as shPfkp-2 and shPfkp-5, respectively. The puromycin-resistance gene was excised with restriction enzymes, and the hygromycin-resistance gene was ligated into the same region of each vector.

### Capillary electrophoresis-mass spectrometry (CE-MS)

Organoids were collected through complete dilution of gels by Cell Recovery Solution (BD Bioscience, San Jose, CA, USA) and washed twice with 5% mannitol in order to extract the metabolites. Methanol containing 25 μM each of methionine sulfone, 2-(N-morpholino)ethanesulfonic acid (MES), and (+)-Camphor-10-sulfonic acid was added to cell pellets, which were left to rest for 10 min at room temperature. Samples in solution were mixed with water and chloroform, centrifuged at 10,000 × *g* for 3 min at 4 °C, and the aqueous phase of the solution was removed. The extract was transferred to a 5 kDa ultrafiltration tube (Human Metabolome Technologies, Yamagata, Japan) and centrifuged at 9,100 × *g* for 2 h at 20 °C. CE-MS was used to analyze the filtrate in Dr. Soga’s laboratory, as previously described^50^.

### Transcriptional analysis

GENE CHIP Expression Analysis microarrays were performed following the manufacturer’s protocol (Affymetrix, Santa Clara, CA, USA). Total RNA was extracted from murine liver organoids expressing human wild type IDH1 and mutant IDH1 (R132C), at passage three after transfection.

### Animals

All experiments were performed under protocols approved by the Animal Ethics Committee of The University of Tokyo. The research was conducted with guidelines approved by the Office of Laboratory Animal Welfare (Ref: F18-00412). C57BL/6 J mice were purchased from CLEA (Shizuoka, Japan).

### Products

AGI-5198 was purchased from Cayman Chemical (Ann Arbor, MI). R(–)-2-hydroxyglutarate (2-HG) and 2-deoxy-d-glucose (2DG) were purchased from Sigma-Aldrich. Cell Titer-Glo was purchased from Promega (Madison, WI). Compound C (ab146597) was purchased from Abcam (Cambridge, UK). DMEM/F12 without Glucose (L0091) was purchased from Biowest (Nuaille, France).

### Clinical samples, molecular analysis, and PFKP scoring system

Formalin-fixed, paraffin-embedded (FFPE) tissue blocks from 101 patients with ICC who underwent surgical treatment at the University of Tokyo Hospital were used for the extraction of tumor DNA. Mutational analysis of *IDH* was performed by direct sequencing of PCR products. FFPE tissue sections were immunohistochemically stained using the primary antibody against PFKP (ab119796, Abcam). Staining results were scored by two pathologists (K.M. and A.H.) blinded to the mutational profile of *IDH*. The scoring system was validated by the percentages of positive cells as follows: score 0, <1%; score 1, 1–5%; score 2, 6–25%; score 3, 26–50%; and score 4, >51%. Simultaneously, scores 0 and 1 were defined as negative expression, score 2 as low, score 3 as moderate, and score 4 was defined as high expression, respectively. Detailed procedures for immunohistochemistry and DNA sequencing were previously described^51^. This study was approved by the Ethics Committee of the University of Tokyo, and carried out in accordance with the Declaration of Helsinki. Informed consent in writing was obtained from all patients.

### PFK-1 activity assay

6-Phosphofructokinase Activity Assay Kit (ab155898, Abcam) was used in accordance with the manufacturer’s protocol. Briefly, for each assay, 900,000 cells were harvested and washed in cold PBS. After suspension and appropriate dilution in the assay buffer, cellular extracts were mixed with the reaction solution containing ATP and F6P. NADH produced by the reaction was monitored spectrophotometrically at 450 nm for 100 min. PFK-1 activity was calculated from an NADH standard curve.

### Glucose uptake assay

Glucose Uptake Assay Kit (ab136955, Abcam) was used in accordance with the manufacturer’s protocol. Briefly, organoids were treated in Krebs-Ringer-Phosphate-Hepes (KRPH) buffer: 20 mM HEPES, 5 mM KH2PO4, 1 mM MgSO4, 1 mM CaCl2, 136 mM NaCl, and 4.7 mM KCl, at a pH 7.4 containing 2% bovine serum albumin (BSA), with or without 2-DG (1 mM), for 20 min. Organoids were harvested and washed in cold PBS. For each assay, we used 70,000 cells. Harvested cells were lysed with extraction buffer, frozen at −80 °C for 15 min, and heated at 85 °C for 40 min. After cooling on ice for 5 min, the lysates were neutralized by adding neutralization buffer and diluted appropriately with assay buffer. Lastly, by two amplification steps, according to the manufacturer’s instructions, the colorimetric end product was detected at 412 nm. From the 2-DG-6-phosphate (2-DG6P) standard curve, 2-DG (or glucose) uptake was calculated.

### Quantitative real-time reverse transcriptase-PCR (RT-qPCR)

RT-qPCR was performed as previously described^52^. RNA was extracted from organoids using RNeasy Mini Kit (Qiagen, Hilden, Germany). Values were internally normalized against β-actin (*Actb*) mRNA expression. PCR primer sequences are listed in Supplementary Table 2.

### Immunohistochemistry

Organoids were fixed in 4% paraformaldehyde (PFA) in PBS. The slides were hematoxylin & eosin (H&E) stained and subjected to histological analysis. Immunohistochemistry was performed as previously described^52^. The primary antibodies used were anti-Ck19 (DSHB, AB-2133570, 1:100) and anti-Sox9 (Sigma-Aldrich, AB5535, 1:1000).

### Immunoblotting

Harvested organoids were lysed in RIPA buffer [10 mM Tris-HCl (pH 7.4)], 150 mM NaCl, 2 mM EDTA, 1% NP40, 0.1% Na deoxycholate, 0.1% SDS, 50 mM NaF, 1 mM Na3VO4, and a protease inhibitor cocktail (Complete Mini, Roche, Basel, Switzerland), then sonicated for 5 min. After centrifugation at 15,000 × *g* for 15 min, supernatants were collected as whole cell lysates. Immunoblotting was performed as previously described^52^. The primary antibodies used were anti-β-actin (Sigma-Aldrich, A5441, 1:10,000), anti-Akt (Cell Signaling, 9272, 1:1,000), anti-phosphorylated-Akt (Cell Signaling, 9271, 1:1,000), anti-Histone H3 (Abcam, ab1791, 1:1,000), anti-H3K9me3 (Abcam, ab8898, 1:1,000), anti-H3K9me1 (Abcam, ab9045, 1:1,000), anti-H3K4me3 (Abcam, ab8580, 1:1,000), anti-H3K27me3 (Abcam, ab6002, 1:1,000), anti-Pfkp (Abcam, ab119796, 1:500), anti-Pfkl (Abcam, ab37583, 1:500), anti-Pfkm (Proteintech, 55028-1-AP, 1:800), anti-Erk (Cell Signaling, 9102, 1:1000), anti- phosphorylated Erk (Cell Signaling, 9101, 1:1000), anti-Ampk (Cell Signaling, 2532, 1:1000), anti- phosphorylated Ampk (Cell Signaling, 2535, 1:1000), anti-Hif-1α (Abcam, ab463, 1:1000), anti-Lkb1 (Cell Signaling, 3047, 1:1000), anti-phosphorylated-Lkb1 (Cell Signaling, 3482, 1:1000), anti-Camkk2 (Cell Signaling, 16810, 1:1000), anti-phosphorylated-Camkk2 (Cell Signaling, 12818, 1:1000), anti-Pfkfb3 (Abcam, ab181861, 1:1000), anti-phosphorylated-Pfkfb3 (Abcam, ab202291, 1:1000), anti-p70S6k (Cell Signaling, 2708, 1:1000), anti-phosphorylated-p70S6k (Cell Signaling, 9205, 1:1000), anti-Ulk1 (Cell Signaling, 8054, 1:1000), anti-phosphorylated-Ulk1 (Cell Signaling, 5869, 1:1000), anti-Beclin1 (Cell Signaling, 3495, 1:1000), anti-phosphorylated-Beclin1 (Cell Signaling, 14717, 1:1000), anti-Raptor (Cell Signaling, 2280, 1:1000), anti- phosphorylated- Raptor (Cell Signaling, 2083, 1:1000), anti-ACC (Cell Signaling, 3676, 1:1000), and anti- phosphorylated- ACC (Cell Signaling, 11818, 1:1000).

### Dot blot analysis

Genomic DNA samples were collected from organoids using QIAamp DNA Mini Kit (Qiagen). Dot blot analysis was performed as described previously^53^. The primary antibodies used were anti-5-hydroxymethylcytosine (5-hmC) (39769, 1:10,000, Active Motif, Carlsbad, CA, USA) and anti-5-Methylcytosine (5-mC) (NA81, 1:1,000, Merck Millipore, Burlington, MA, USA).

### ATAC-seq

ATAC-seq experiments were performed on 100,000 cells from the organoid samples, following published protocols^27^. We prepared the samples from wt-/mut-IBOs, at passage 8. Processing of ATAC-Seq reads were performed as follows. Sequence reads were trimmed and aligned to the mouse genome (mm10) using BWA^54^. PCR duplicates were removed using Picard’s MarkDuplicates, and peaks were called using MACS2^55^. Called peaks were further processed to obtain scatter-plots, correlation heat maps, and Venn diagrams using DiffBind^27^.

### Chromatin immunoprecipitation (ChIP)

ChIP was performed as previously described^52,56^. We prepared 1.5 × 10^6^ cells from liver organoids expressing human wild type IDH1 and mutant IDH1 for each assay. Antibodies used for ChIP were H3K4me3 (ab8580, Abcam) and normal Rabbit IgG (CST2729, Cell Signaling Technology, Danvers, MA). ChIP primers are listed in Supplementary Table 3.

### Statistical analysis

Data are presented as means ± standard error of the mean (SEM). Statistical significance was evaluated by the two-tailed Student’s *t*-test. * denotes P < 0.05, and **P < 0.01. To test the correlation between PFKP expression and IDH status in human ICC samples, Fisher’s exact test was used.

## Supplementary information

Supplementary information

Supplementary information 2

Supplementary information 3

## Data Availability

The datasets analyzed in the current study are available from the corresponding author on reasonable request. Microarray gene expression data are in Gene Expression Omnibus (GSE134760).
