# Supplementary material for: Mutant IDH1 confers resistance to energy stress in normal biliary cells through PFKP-induced aerobic glycolysis and AMPK activation
Source: Sci Rep. 2019 Dec 11;9:18859. doi: 10.1038/s41598-019-55211-w (PMC6906335; doi:10.1038/s41598-019-55211-w)

Mutant IDH1 confers resistance to energy stress in normal biliary cells through PFKP-induced aerobic glycolysis and AMPK activation

Author names

Hiroaki Fujiwara[1,2], Keisuke Tateishi*[1], Kento Misumi[3], Akimasa Hayashi[3], Kaori Igarashi[4], Hiroyuki Kato[1], Takuma Nakatsuka[1], Nobumi Suzuki[1], Keisuke Yamamoto[1], Yotaro Kudo[1], Yoku Hayakawa[1], Hayato Nakagawa[1], Yasuo Tanaka[1], Hideaki Ijichi[1], Hirofumi Kogure[1], Yosuke Nakai[1], Hiroyuki Isayama[6], Kiyoshi Hasegawa[5], Masashi Fukayama[3], Tomoyoshi Soga[4] and Kazuhiko Koike[1]

Affiliations

[1]Department of Gastroenterology, Graduate School of Medicine, The University of Tokyo, Tokyo, Japan

[2]Division of Gastroenterology, The Institute for Adult Diseases, Asahi Life Foundation, Tokyo, Japan.

[3]Department of Pathology, Graduate School of Medicine, The University of Tokyo, Tokyo, Japan.

[4]Institute for Advanced Biosciences, Keio University, Yamagata, Japan.

[5]Hepato-Biliary-Pancreatic Division, Department of Surgery, Graduate School of Medicine, The University of Tokyo, Tokyo, Japan.

[6]Department of Gastroenterology, Graduate School of Medicine, Juntendo University, Tokyo, Japan.

Supplementary Figure 1-9

Supplementary Table 1-3

Uncropped images of western blots for main figures

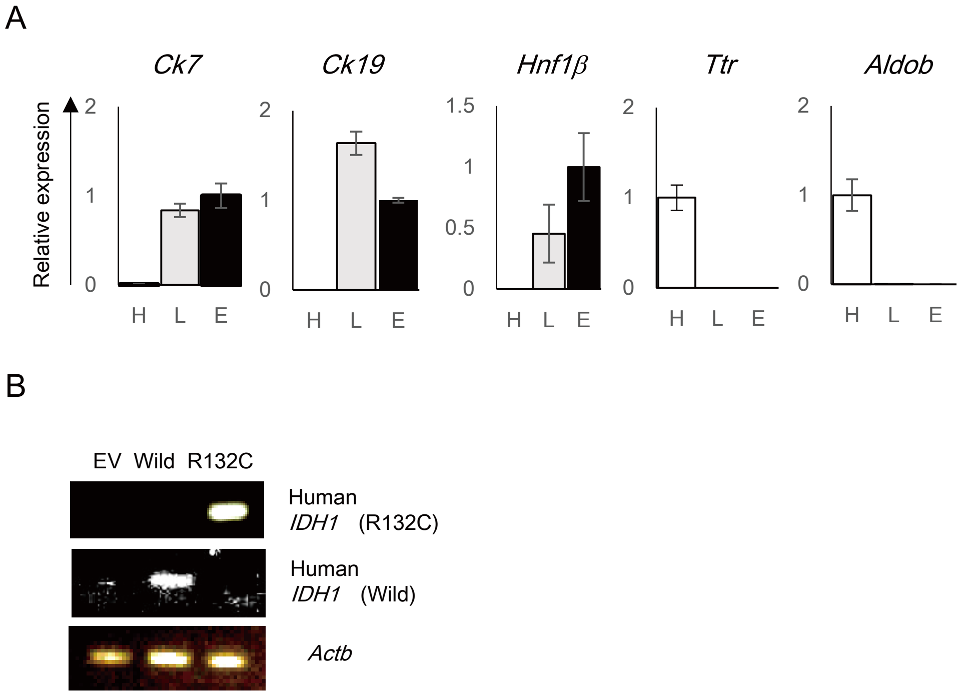

Supplementary Figure 1. Establishment of IBOs stably expressing wild-type and mutant IDH1. (A) Gene expression levels of biliary and hepatocyte markers by RT-qPCR. H, hepatocytes; L, organoids from murine livers; E, organoids from extrahepatic bile ducts. (B) Validation of human *IDH1* mRNA expression in IBOs stably expressing empty vector (EV), wild-type (Wild), and mutant IDH1 (R132C) by RT-PCR using wild-type or mutant IDH1-specific primers.

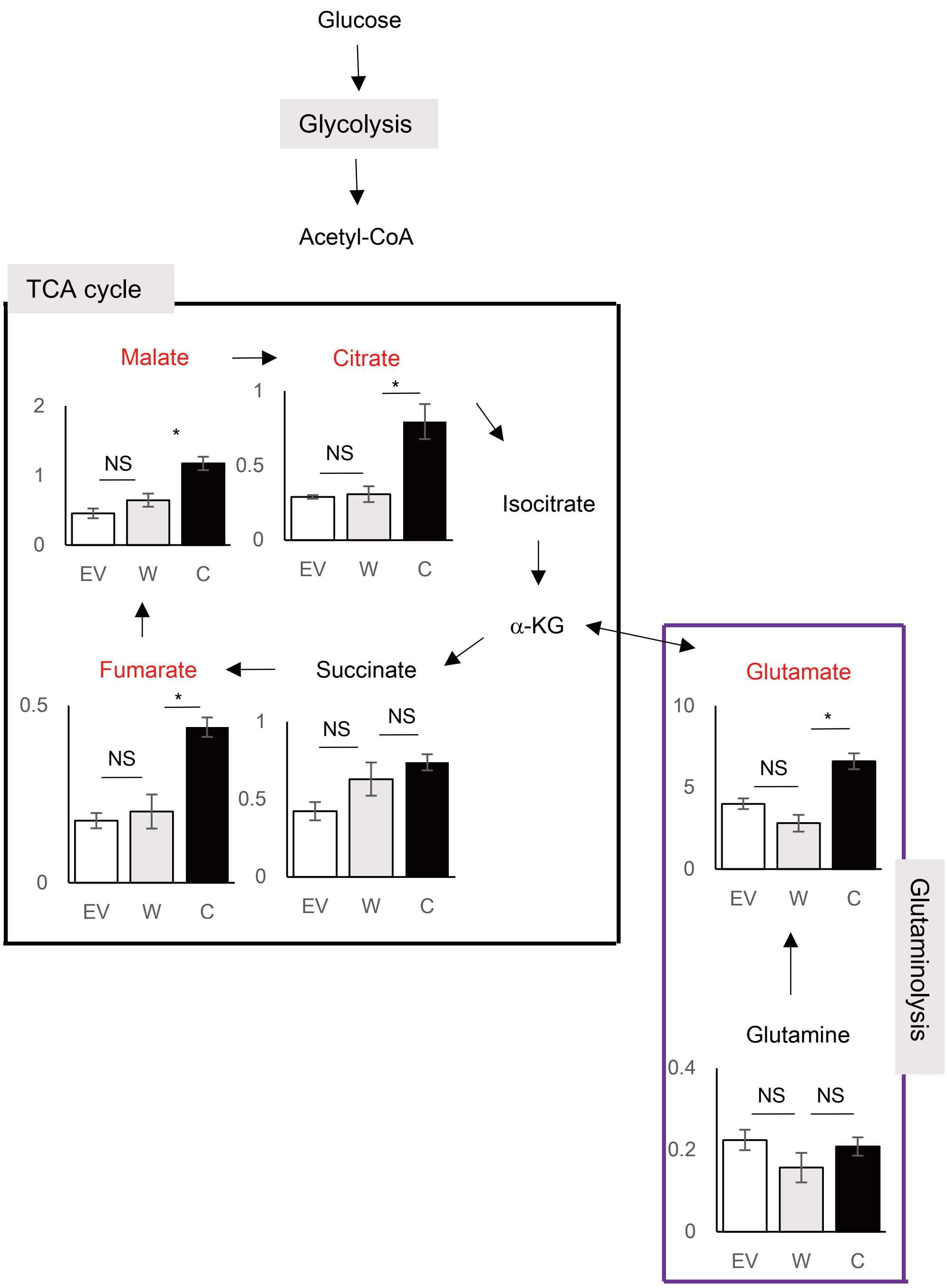

Supplementary Figure 2. Intermediates of TCA cycle and glutaminolysis in IBOs. Metabolomic analysis of IBOs stably expressing empty vector (EV), wild-type IDH1 (Wild) or mutant IDH1 (R132C) by CE-MS (n = 4, * P < 0.05, NS not significant).

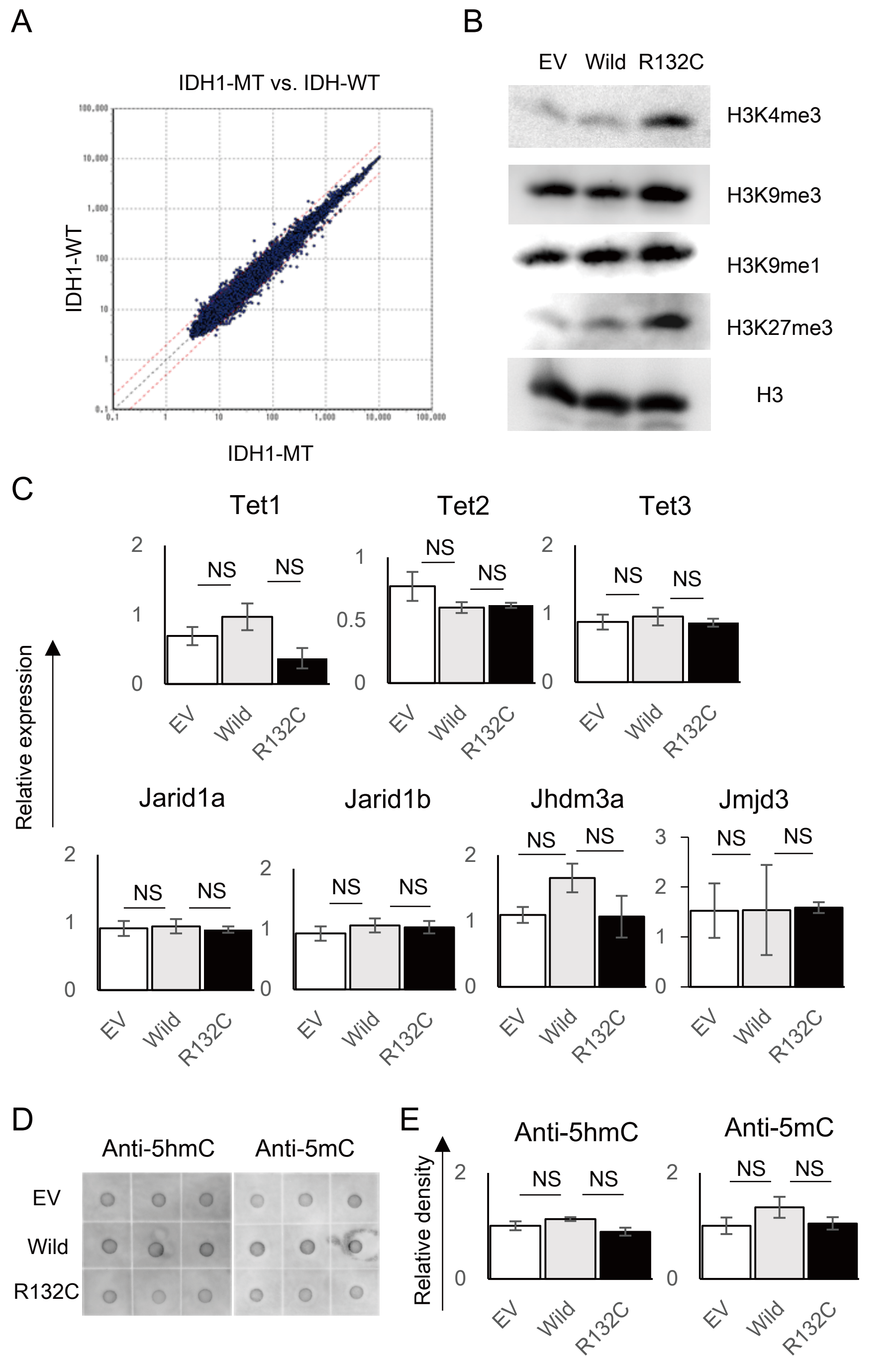

Supplementary Figure 3. Epigenetic status of IBOs. (A) Scatter plot of microarray data of wt- and mut-IBOs. Red dotted lines indicate a two-fold increase or 50% decrease in expression levels. (B) Global histone methylation assessed by western blotting with specific antibodies. (C) Relative gene expression levels of Tet family genes and α-KG dependent histone demethylases (n = 4, * $P < 0.05$, NS not significant). (D, E) Dot blot analysis of 5-mC and 5-hmC (D), and densitometric analysis (E).

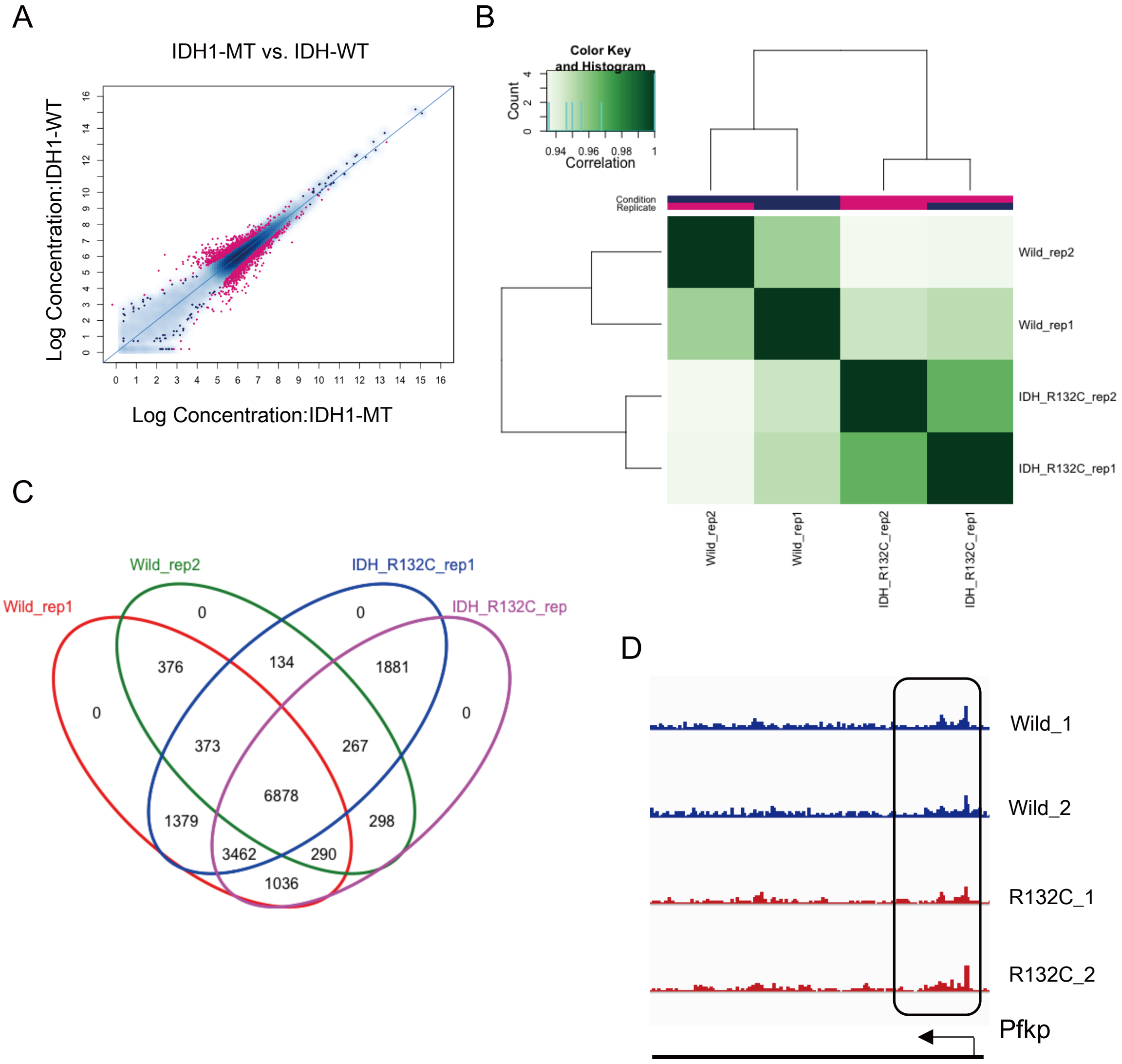

Supplementary Figure 4. ATAC-seq analysis of IBOs. (A) Scatter plots of ATAC-seq peaks comparing samples from IBOs expressing the indicated *IDH* allele. (B) Clustering of Pearson's correlations of ATAC-seq peaks between samples (Wild_rep1 and its replicate Wild_rep2, IDH1_R132C_rep1 and its replicate IDH1_R132C_rep2). (C) Venn diagram demonstrating the intersection of wild-type (Wild) and mutant (R132C) IDH1-IBOs peaks. (D) ATAC-seq data from IBOs expressing wild-type (Wild_1, 2) and mutant IDH1 (R132C_1, 2) in the promoter region of *Pfkp*.

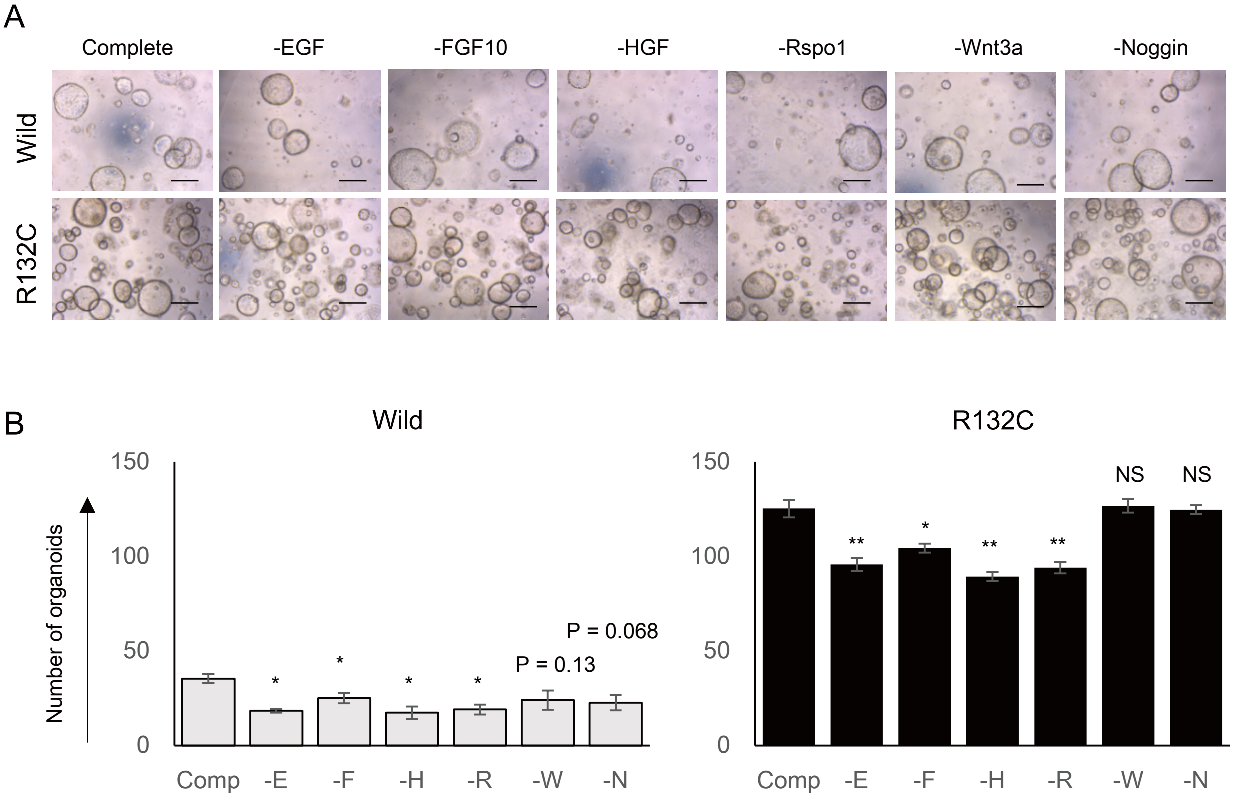

Supplementary Figure 5. IDH1 mutation affects niche dependency in IBOs. (A) Representative images of IBOs under each condition. (B) The number of organoids (> 100 μm) at day 5 (n = 3, * P < 0.05, ** P < 0.01, NS not significant). Scale bars, 500 μm.

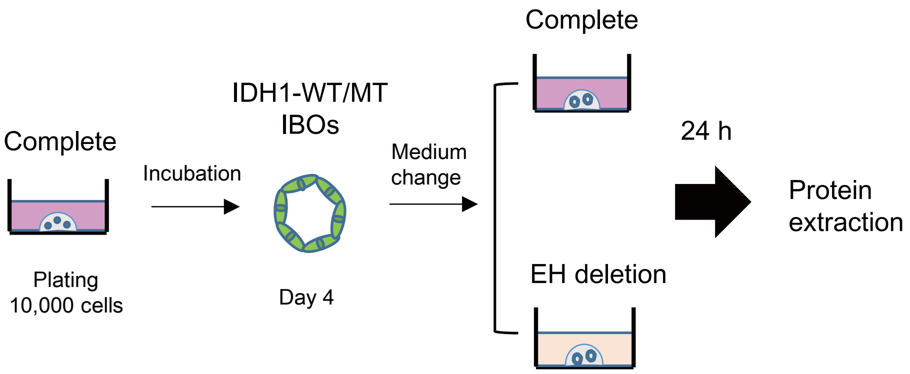

Supplementary Figure 6. Experimental design validating the effect of EH deletion in protein expression of IBOs. IBOs expressing wild-type and mutant IDH1 were incubated for 4 days in complete medium, and proteins were extracted 24 h after changing complete medium (Complete) or medium without EGF and HGF (EH deletion).

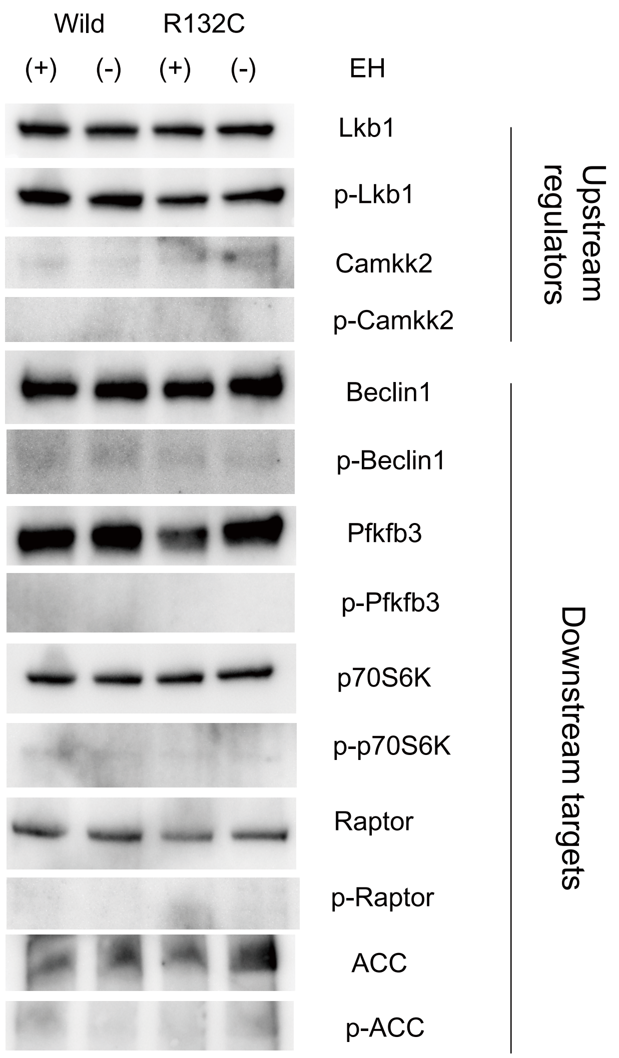

Supplementary figure 7. Upstream regulators and downstream targets of the AMPK signaling pathway in IBOs. Immunoblots for Lkb1, phosphorylated Lkb1 (p-Lkb1), Camkk2, phosphorylated Camkk2 (p-Camkk2), Beclin1, phosphorylated-Beclin1 (p-Beclin1), Pfkfb3, phosphorylated-Pfkfb3 (p-Pfkfb3), p70S6K, phosphorylated-p70S6K (p-p70S6K), Raptor, phosphorylated-Raptor (p-Raptor), ACC, and phosphorylated-ACC (p-ACC) in IBOs expressing wild-type and mutant IDH1. Each sample was extracted as shown in Supplementary Fig. 6.

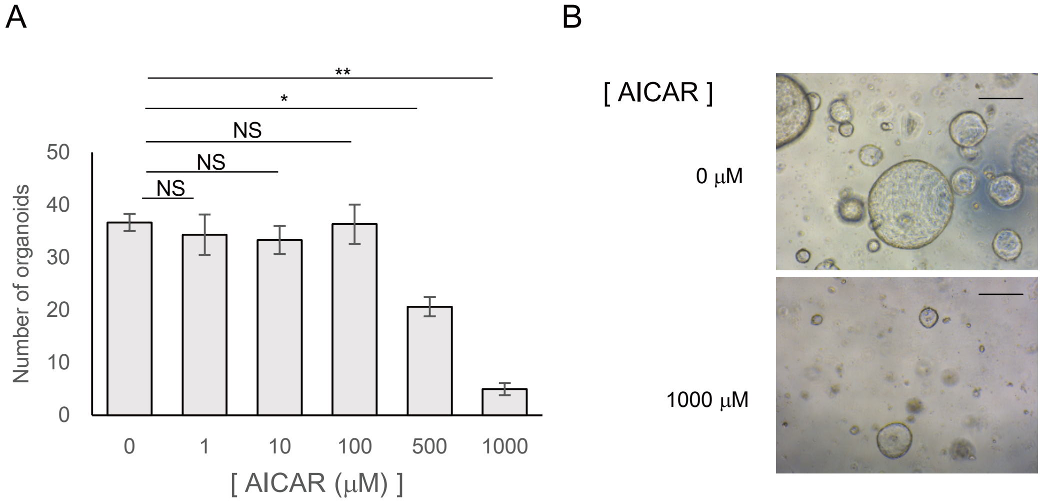

Supplementary Figure 8. AICAR treatment decreases the formation of IBOs.

(A, B) Dissociated wild-type IDH1 IBO cells treated with vehicle or indicated doses of AICAR (10,000 cells per group). (A) The number of organoids (> 100 μm) at day 5 (n = 3, * P < 0.05, ** P < 0.01, NS not significant). (B) Representative images. Scale bars, 500 μm.

A

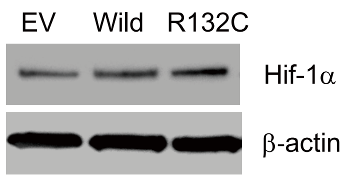

B

Gene symbol	Fold-change (IDH1-MT/WT)	p-value
Glut1	1.439914874	0.018546
Glut2	0.86152606	0.443318
Glut3	1.053541382	0.083464
Glut4	0.804497719	0.223257
Hk1	1.274316384	0.000809
Hk2	1.198663334	0.003766
Hk3	0.962318795	0.679952
HK4	0.960290674	0.776537
Pfkfb1	0.894617551	0.072145
Pfkfb2	1.27057319	0.048331
Pfkfb3	1.229288685	0.000687
Pfkfb4	0.944468025	0.2188
Aldoa	1.276805849	0.372986922
Aldob	0.828784039	0.003306251
Gapdh	1.120045289	0.054044993
Pgk1	1.386864079	0.009007
Pgam1	1.137919054	0.224605309
Eno1	1.460588261	0.2033705
Pkm	1.104129065	0.11327
Pdk1	1.481089699	0.025833
Pdk2	1.32846288	0.00551
Pdk3	0.899855848	0.262461
Pdk4	0.893940276	0.145724
Ldha	1.203309185	0.091143
Ldhb	0.803689927	0.164615704

Supplementary Figure 9. IDH1 mutation does not markedly alter protein levels of Hif-1α and the expression of its target glycolytic genes in IBOs

(A) Protein expression levels of Hif1α in IBOs expressing empty vector (EV), wild-type (Wild) or mutant IDH1 (R132C). (B) Results of cDNA microarray analysis regarding Hif-1α target glycolytic gene expression in IBOs expressing wild-type and mutant IDH1.

Supplementary Table 1. The candidate target genes for IDH1 mutation in IBOs

A

Ratio {IDH1-MT/WT}	P-value	Gene Symbol	Gene Description
4.118727052	1.48E-05	Aqp1	aquaporin 1
3.434380045	0.019354557	Cfh	complement component factor h
3.12805744	0.002315042	Cyp2s1	cytochrome P450, family 2, subfamily s, polypeptide 1
3.084746214	0.002946664	AA467197	expressed sequence AA467197
2.497245087	0.001066294	Serpine1	serine (or cysteine) peptidase inhibitor, clade E, member 1
2.487374761	0.004799826	Ndrg1	N-myc downstream regulated gene 1
2.415147159	0.001294176	1810008I18Rik	RIKEN cDNA 1810008I18 gene
2.413548548	0.003119969	Epas1	endothelial PAS domain protein 1
2.288530138	0.003595424	Bnip3	BCL2/adenovirus E1B interacting protein 3
2.204185826	0.008994044	Ankrd37	ankyrin repeat domain 37
2.144644689	0.010116781	Ero1l	ERO1-like (S. cerevisiae)
2.127769952	0.013883511	Selenbp1	selenium binding protein 1
2.106117663	0.011928974	Id3	inhibitor of DNA binding 3
2.099093733	0.006649894	Hgfac	hepatocyte growth factor activator
2.09896057	0.00774717	Spink4	serine peptidase inhibitor, Kazal type 4
2.097151284	0.014436165	Ak4	adenylate kinase 4
2.094589175	0.004059593	AU021092	expressed sequence AU021092
2.074856812	0.00061282	Gm3336	predicted gene 3336
2.066912977	0.015759457	Bst1	bone marrow stromal cell antigen 1
2.01051542	0.001504503	Pfkp	phosphofructokinase, platelet

B

Ratio {IDH1-MT/WT}	P-value	Gene Symbol	Gene Description
0.198005982	0.000532654	Fn1	fibronectin 1
0.220566264	0.00373881	Vcam1	vascular cell adhesion molecule 1
0.337010193	0.038075721	Shisa6	shisa homolog 6 (Xenopus laevis)
0.339115219	0.001474066	Ctse	cathepsin E
0.368310724	0.000408695	Tnfrsf11b	tumor necrosis factor receptor superfamily, member 11b (osteoprotegerin)
0.374496986	0.018121948	Pamr1	peptidase domain containing associated with muscle regeneration 1
0.396913195	0.0000911	Ighd6-2	immunoglobulin heavy diversity 6-2
0.420286068	0.006792437	Tlcd2	TLC domain containing 2
0.429650441	0.032118843	Rec8	REC8 meiotic recombination protein
0.433642757	0.006193068	Pik3r3	phosphatidylinositol 3 kinase, regulatory subunit, polypeptide 3 (p55)
0.43548875	0.0034724	Clic6	chloride intracellular channel 6
0.438751454	0.000303343	Ankrd1	ankyrin repeat domain 1 (cardiac muscle)
0.445183519	0.015095075	Serpinb9	serine (or cysteine) peptidase inhibitor, clade B, member 9
0.449102162	0.031463259	Cdh6	cadherin 6
0.45672529	0.081245334	Gm17332	predicted gene, 17332
0.459824824	0.000964351	Ptprb	protein tyrosine phosphatase, receptor type, B
0.463824419	0.000805122	Egfl6	EGF-like-domain, multiple 6
0.471155312	0.021254851	Zdhhc15	zinc finger, DHHC domain containing 15
0.477807111	0.006964567	Soga3	SOGA family member 3
0.482353237	0.005732729	Pxdn	peroxidasin homolog (Drosophila)
0.486656373	0.011098523	Tm4sf4	transmembrane 4 superfamily member 4
0.486750122	0.004612527	Igfbp7	insulin-like growth factor binding protein 7
0.488978372	0.033019457	Il1a	interleukin 1 alpha
0.490340524	0.007071928	Plet1	placenta expressed transcript 1
0.493846576	0.042502858	Faxc	failed axon connections homolog (Drosophila)
0.494504756	0.023286157	Trp53i11	transformation related protein 53 inducible protein 11
0.49687905	0.032505462	Cd63	CD63 antigen

Results of cDNA microarray analysis showing the candidate genes differentially upregulated (A) and downregulated (B) between IBOs stably expressing wild-type (WT) and mutant IDH1 (MT).

Supplementary table 2. Lists of primers for RT-qPCR

Target gene	Forward primer (5' to 3')	Reverse primer (5' to 3')
Pfkp	GAGCACCGTCTCCATTCGAT	CCCTTCAGTTTGGCCGAGAT
Ck7	GTTTCAGACTATCTTCCAGG	TATCTCTGTTGTGAATTCCA
Ck19	CGGTGGAAGTTTTAGTGGGA	AGTAGGAGGCGAGACGATCA
Hnf1β	AGATCACAGTGTCGGGAGGA	GGAGGTGTTGAGGCTCTGTG
Ttr	CACCAAATCGTACTGGAAGACA	GTCGTTGGCTGTGAAAACCAC
Aldob	TGTCTGGAGGTATGAGTGAGG	CTGGGTTGCCTTCTTGTTTGC
Tet1	TCTCTCATAGAGTGAGCTAGCACGTAA	GCCCGCTGAGTCCTGTAAAC
Tet2	GTGGGAGGCAGATCTGTGAT	GTTGACACCAAGCAGAAGCA
Tet3	GGGAGACCTCAGCACAGAAG	ATAGAAGCGAACGAGGAGCA
Jarid1a	CCTCCATTTGCCTGTGAAGT	CCTTTGCTGGCAACAATCTT
Jarid1b	AGAGGCTGAATGAGCTGGAG	TGGCAATTTTGGTCCATTTT
Jhdm3a	GACCACACTCTGCCCACAC	TCCTGGGGTATTTCCAGACA
Jmjd3	CCCCCATTTCAGCTGACTAA	CTGGACCAAGGGGTGTGTT
Actb	ATGTGGATCAGCAAGCAGGA	AAGGGTGTAAAACGCAGCTCA

Supplementary table 3. ChIP-qPCR primers

Gene	Forward primer (5' to 3')	Reverse primer (5' to 3')	Target site
Pfkp	CCTCTGTTCCCAACCCGATT	GGGATGGCAAAGCTATCGGT	200bp upstream of TSS
Pfkp	CATTGCGGATGGCAAGTCTG	AGCGTCCGTGCTGTATTTGA	50kbp upstream of TSS

Primer pairs were designed to recognize about 500bp sequence upstream of the transcription start site (TSS) or the region 50kbp upstream of TSS in *Pfkp* gene.

Uncropped images of western blots for main figures

Figure 4B

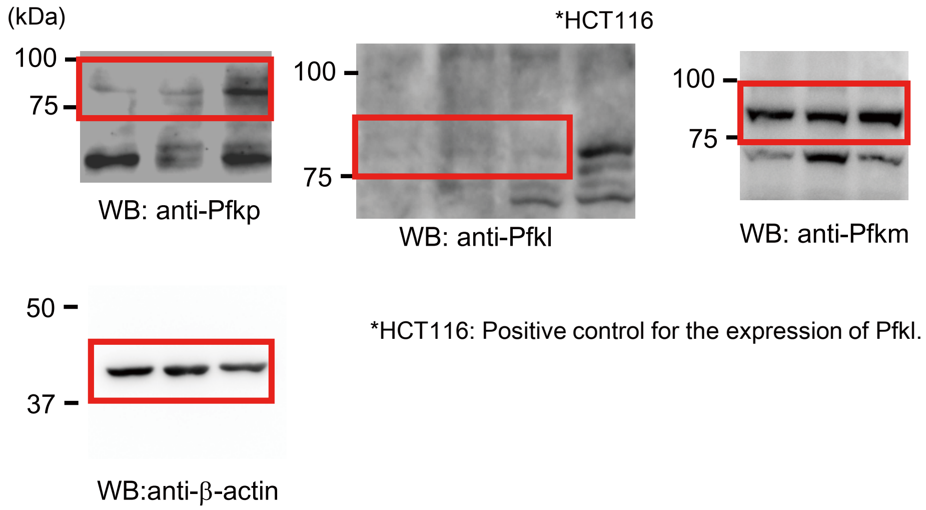

Figure 7A

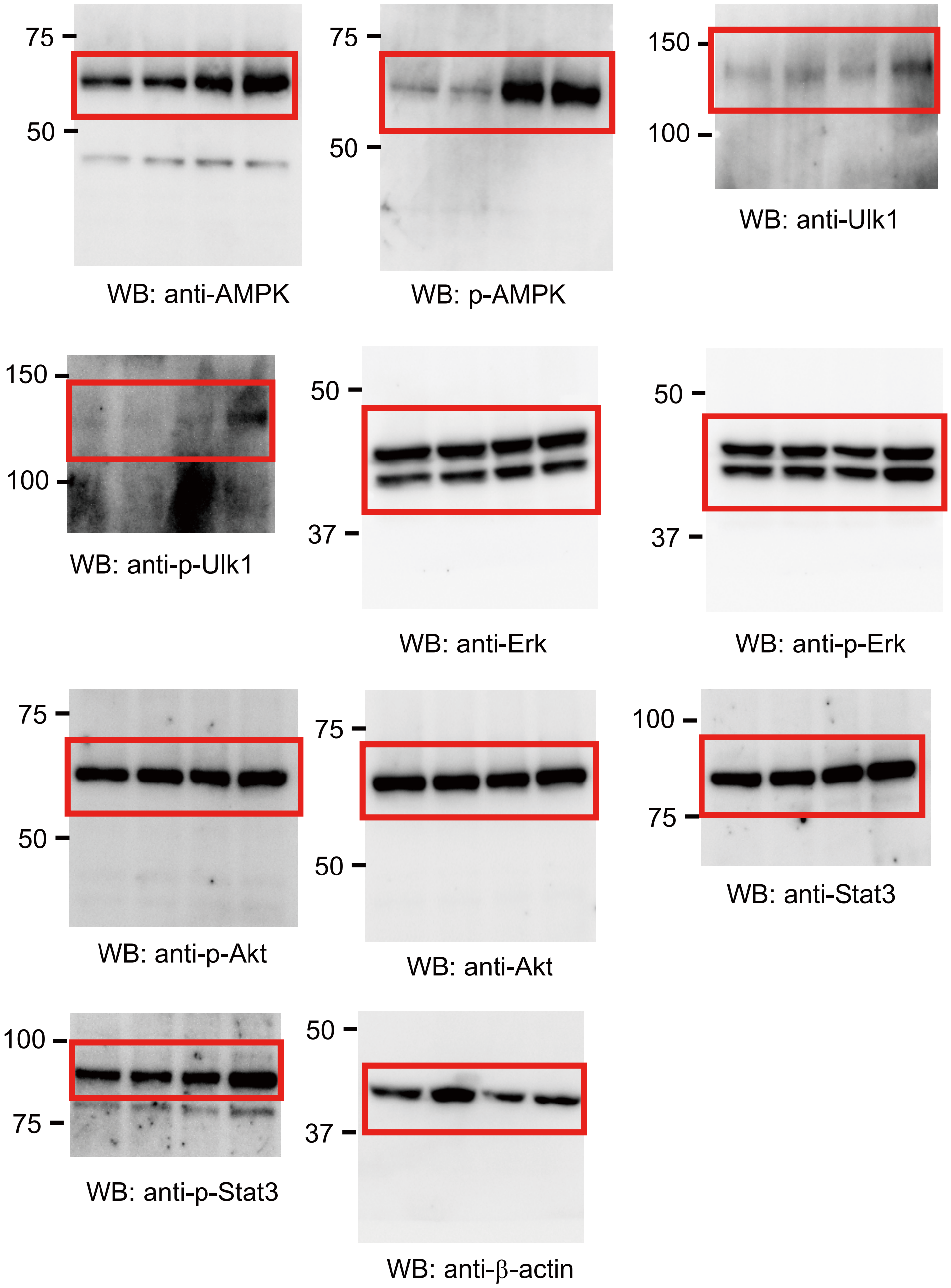

Supplement: Supplementary file 3 — Supplementary information 3 [file 41598_2019_55211_MOESM3_ESM.pdf]
